# Exploring the relationship between mental work load, work ability, and musculoskeletal disorders: A cross-sectional modeling study among health service workforces in Iran

**DOI:** 10.1371/journal.pone.0322993

**Published:** 2025-05-13

**Authors:** Maryam Rafiee, Ali Alboghobeish, Mahnaz Saremi, Sayed Vahid Esmaeili

**Affiliations:** 1 Student Research Committee, Department of Ergonomics, School of Public Health and Safety, Shahid Beheshti University of Medical Sciences, Tehran, Iran; 2 Student Research Committee, Department of Occupational Health and Safety Engineering, School of Public Health and Safety, Shahid Beheshti University of Medical Sciences, Tehran, Iran; 3 Department of HSE, Marun Petrochemical Company, Bandare-Mahshahr, Iran; 4 Department of Ergonomics, School of Public Health and Safety, Shahid Beheshti University of Medical Sciences, Tehran, Iran; Nnamdi Azikiwe University, NIGERIA

## Abstract

**Introduction:**

The health service workforces play a crucial role in shaping a nation’s health system efficiency, making it vital to understand factors affecting their performance. This study examined the relationship between mental workload, work ability, and work-related musculoskeletal disorders (WMSDs) among employees at comprehensive health service centers in Saveh, Arak province, Iran.

**Materials & Methods:**

The cross-sectional study involved 197 randomly selected personnel whose WMSDs prevalence was evaluated using the Nordic questionnaire. Work ability was assessed through the Work Ability Index (WAI), and mental workload was measured using the NASA Task Load Index (NASA-TLX). All data analysis was performed using SPSS v 24.0 software.

**Results:**

The average age and work experience of the participants were determined to be 35.74 ± 7.52 and 11.63 ± 8.59 years, respectively. The highest prevalence was reported in the lumbar region, with a lifetime prevalence of 63.5% and an annual prevalence of 60.9%. The average mental workload and work ability were calculated to be 63.24 ± 13.26 and 34.86 ± 7.61, respectively. The highest prevalence of WMSDs (89.5%) was observed in the lower back region among women aged 45–54 years. A significant relationship was identified between work experience and age with work ability and mental workload (P < 0/01). Furthermore, an increase of one point in work ability score leads to a decrease in WMSDs in the neck, wrists/hands, low back and hips/thighs regions by 13.5%, 8%, 11.5%, and 9%, respectively.

**Conclusions:**

The study underscores the need to enhance the physical, psychological, and social environments of health service employees. Implementing targeted educational programs can improve task performance and mitigate high mental workload effects, ultimately reducing WMSDs and improving staff well-being.

## 1. Introduction

In today’s rapidly evolving healthcare landscape, providing services to people in remote areas has become a cornerstone of achieving equitable health outcomes [1]. Comprehensive health centers play an important role in bridging the gap between access to healthcare and the basic needs of underserved populations [2]. These centers are often staffed by dedicated professionals who, despite numerous challenges, work tirelessly to ensure that people in remote communities receive quality healthcare [3]. The importance of these services cannot be overstated, as they not only improve the health and well-being of the population, but also contribute to the overall stability and growth of such communities [4].

However, staff working in these facilities often face a variety of challenges that can significantly affect their health and well-being [5]. The demanding nature of healthcare work, particularly in remote areas, often leads to increased mental stress, which in turn can affect the physical health of staff [6]. Healthcare workers, including nurses, paramedics and community health workers, are particularly vulnerable to ill health caused by prolonged mental stress [7]. Statistics show that mental health problems, work-related stress and work-related musculoskeletal disorders (WMSDs) are widespread in these professions, posing a significant risk not only to the individuals concerned but also to the overall efficiency of the healthcare services provided [8].

Given these challenges, it is important to examine the multifaceted relationship between mental workload, work ability and the incidence of WMSDs [9]. Mental workload refers to the amount of cognitive effort invested in performing tasks, which can vary greatly among healthcare professionals depending on various factors such as patient load, administrative tasks and emotional stress [10]. At the same time, work ability reflects a person’s ability to cope with work demands, taking into account the person’s health status, physical fitness and motivation [11]. By understanding how these variables interact, we can gain important insights into the health and sustainability of the health service workforces.

Furthermore, the backdrop against which this research takes place underscores the urgent need to address the health of the health service workforces. The reported prevalence of WMSDs in this population has reached alarming levels, with studies suggesting that they are a leading cause of work-related disability in healthcare professionals [8,12,13]. A comprehensive analysis of how mental workload affects both the ability to work and the physical ailments of these personnel is crucial. This research methodology not only enables a differentiated understanding of the problems at hand, but also highlights the need for targeted interventions.

Previous research has found clear links between chronic stress and physical health, but the specific context of healthcare workers in remote areas has been less explored [14,15]. Most existing studies focus primarily on well-resourced urban health service workforces settings, leaving a notable gap in our understanding of the particular challenges faced by health service workforces in remote and low-resource areas, including small towns and rural areas with limited infrastructure [16]. By focusing on these underrepresented populations, this study aims to fill a critical gap in the existing literature while providing insights for targeted interventions.

The impact of these health challenges goes beyond employee retention and satisfaction; it threatens the quality and consistency of care for vulnerable populations [17]. High levels of stress and burnout typically lead to lower productivity, more absenteeism and higher turnover rates. Consequently, addressing the overlapping issues of mental workload, work ability and WMSDs in these areas is not just an occupational health issue, but a public health imperative that could improve service delivery in remote communities [18].

Given the significant investment healthcare workers make in the well-being of their patients, it is essential to prioritize their health and working conditions equally [19]. The link between mental workload and its impact on work ability needs to be understood so that appropriate organizational support measures can be developed and implemented. A proactive approach to employee well-being not only promotes individual health, but also improves overall team dynamics and service [20,21].

Given the particular stressors faced by healthcare workers, the exploratory nature of this study is crucial [22]. By using a cross-sectional study design, the study will provide a snapshot of the current state of affairs from which practitioners, managers and policy makers can derive actionable insights. This approach will enable the identification of patterns and correlations, providing the basis for subsequent longitudinal studies or interventions aimed at mitigating the problems identified.

This study aims to illuminate the challenges faced by healthcare workers by examining the relationships between mental workload, work ability, and WMSDs. It advocates for significant changes in workplace policies and practices, emphasizing the importance of addressing workforce health to maintain resilience and effectiveness in healthcare, especially in remote and underserved areas. High mental workload and exceeding work capacity can jeopardize healthcare workers’ health and lead to poor service quality and increased costs for organizations. Therefore, this study specifically investigates the relationship between mental workload, work ability, and WMSD prevalence among employees in both rural and urban comprehensive health service centers located in underserved areas (healthcare access challenges comparable to rural regions due to geographical isolation and resource constraints).

## 2. Methods

### 2.1. Study design and participants

**Fig 1:**
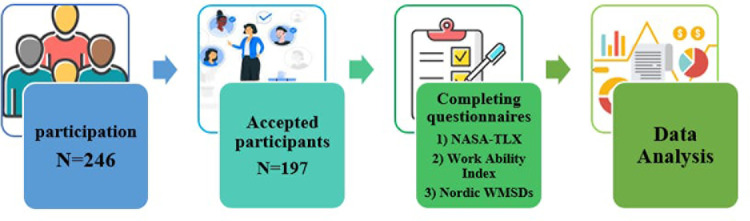
Study flow diagram.

This descriptive-analytical cross-sectional study was conducted on 197 employees of urban and rural comprehensive health service centers located in underserved areas in Saveh, Arak province, Iran, in 2023. To select participants, a list of all health centers and their staff was obtained from the county health department. After determining the sample size, participants were included in the study based on the inclusion criteria, and data were collected using validated tools (See S1 Data). The study’s groupings were based on commonly used age-grouping patterns in WMSDs research. Accordingly, participants’ ages were categorized into four career-stage groups: 20–34 years as young workforce, 35–44 years as experienced workforce, 45–54 years as skilled workforce, and ≥ 55 years as pre-retirement workforce [23]. The protocol for this study was approved by the Medical Ethics Committee of the University of Rehabilitation Sciences, Tehran, Iran. However, all participants completed and approved consent forms. The research proceeded through the steps illustrated in Fig 1.

### 2.2. Sample size calculation

The city of Saveh has 16 urban and 11 rural comprehensive health centers. Among the employees working in these centers, 197 individuals were invited to participate in the study based on random sampling. The sample size was calculated using equation (1) and Table 1).

**Table 1:** Sample size in each occupational subgroup.

Occupation	Population	Sample	Occupation	Population	Sample
Doctor	34	17	Psychologist	10	5
Dentist	27	13	Healthcare worker	135	66
Midwife	28	14	Receptionist	31	15
Healthcare professional	48	24	phlebotomist	14	7
Occupational health expert	13	6	Vaccinator	16	8
Environmental health expert	21	10	Service worker	14	7
Nutritionist	11	5	Total	402	197


$$Equation\;(1):\;n=\frac{N{\left(z_{1-\frac{\alpha }{2}}\right)}^{2}p(1-p)}{d^{2}(N-1)+{\left(z_{1-\frac{\alpha }{2}}\right)}^{2}p(1-p)}=\frac{402(1.\;96{)}^{2}0.\;5(1-0.\;5)}{\left(0.\;05{)}^{2}(402-1)+(1.\;96{)}^{2}0.\;5(1-0.\;5\right)}=197$$

### 2.3. Inclusion and exclusion criteria

Participants in this study were individuals with a single job who had more than one year of work experience and did not have non-WMSDs, such as musculoskeletal injuries due to accidents, sports injuries, lordosis, kyphosis, and scoliosis [24], nor any history of mental illnesses such as depression or behavioral and social interaction imbalances. Participants were able to withdraw from the study at any stage based on their personal decision. Ultimately, failure to complete the questionnaire was also considered as withdrawal from the study.

### 2.4. Data collection tools

To collect the data, a four-part questionnaire was used, which included demographic characteristics, the NASA-TLX questionnaire, the Work Ability Index questionnaire, and the Nordic Musculoskeletal Questionnaire for assessing musculoskeletal disorders:

#### 2.4.1. NASA-TLX questionnaire.

To assess the mental workload of employees, the National Aeronautics and Space Administration Task Load Index (NASA-TLX) was utilized. This tool was developed by the Human Factors Engineering Group at NASA’s Langley Research Center. This method is subjective rating and multidimensional approach that allows for the collection of mental workload scores based on the weighted average of dimensions. The questionnaire consists of six dimensions, each related to an aspect of mental workload:

1. **Mental Demand:** Represents the amount of cognitive and perceptual effort (thinking, decision-making, calculating, recalling, observing, searching, etc.) required to perform the evaluated task.
2. **Physical Demand:** Indicates the physical activity (pushing, pulling, turning, controlling, etc.) necessary to carry out the task under review.
3. **Temporal Demand:** Reflects the time pressure that an individual feels while performing the task.
4. **Performance:** Represents the degree of success in completing the task and the individual’s satisfaction in achieving the expected performance.
5. **Effort:** Indicates the effort required (both physically and mentally) to reach the desired level of efficiency in the task being evaluated.
6. **Frustration:** Reflects the level of feelings of Frustration, disappointment, and stress experienced by the individual during the task.

The assessment of mental workload consists of three sections. In the first section, participants rated each of the six defined dimensions based on their work conditions on a scale from 0 to 100 in 5-point increments (linear scale). In the second section, each dimension was compared pairwise, and the dimension that had a greater impact and importance for the individual or was more related to the activity was selected to determine the frequency of occurrence (workload degree). Then, based on the scores given in the first section, the workload weight was calculated using Equation (2):


Equation (2): workload weight = (scale number−1) * 5

Finally, the total score was calculated using Equation (3):


Equation (3): Total score = (workload degree * workload weight)/ 15

This questionnaire was translated into Persian by Mohammadi et al. and validated on nurses, demonstrating reliability and validity (α = 0.897) [25].

#### 2.4.2. Work ability index.

The Work Ability Index (WAI), designed by the Finnish Institute of Occupational Health in 1981, was utilized to assess work ability [26]. During the 11-year studies conducted by this institute, it was determined that this tool has predictive power regarding individuals’ future disabilities [27]. This index comprises seven dimensions, and the scores for each dimension are calculated as follows [28]:

1. **Current work ability compared to the best period of life:** The worker evaluates their work ability on a scale from zero (completely unable to work) to ten (best work ability throughout life).
2. **Work ability in relation to the physical nature of the job:** A score between 2 and 10 is assigned, ranked from “very poor” to “very good.”
3. **Number of currently diagnosed illnesses by a physician:** In this dimension, the number of illnesses diagnosed by a physician that may affect the individual’s work ability is assessed, with a score ranging from 1 to 7.
4. **Number of work disruptions due to occupational illness:** In this dimension, based on the severity of limitations caused by disorders, a score from 1 to 6 is considered.
5. **Sick leave taken in the past year:** This dimension considers the number of days the individual was unable to work due to illness and thus was on sick leave, with a score ranging from 1 to 5.
6. **Individual’s prediction of their work ability over the next two years:** The score for this dimension ranges from 1 to 7.
7. **Cognitive and mental capabilities:** The minimum score is set at 1, while the maximum score for this dimension is 4.

This index is calculated by summing the scores obtained for each item. The best possible estimate of the index is a score of 49, while the worst estimate is a score of 7. Ultimately, based on the obtained scores, work ability is categorized into four levels: poor (7–27) as “needs immediate intervention”, moderate (28–36) as “needs improvement in work and health status”, good (37–43) as “suitable work ability for the current job”, and excellent (44–49) as “appropriate fit for the job”. Validation of the Persian version of the WAI questionnaire was confirmed by Arastoo et al [29].

#### 2.4.3. Nordic musculoskeletal disorders questionnaire.

The Nordic questionnaire was used to estimate the prevalence of musculoskeletal disorders. This questionnaire was first designed by Kuorina et al. in 1987 [30]. The Nordic questionnaire is intended to assess musculoskeletal problems across a wide range of occupational groups, including industrial workers, nurses, healthcare staff, drivers, and teachers. This tool evaluates the presence of discomfort, burning, or numbness in various body parts, including the neck, shoulders, upper back, elbows, lower back, wrists, thighs, hips, knees, and ankles. The first section assesses discomfort experienced in the past 12 months, the second section covers the past 7 days, and the final section evaluates instances where discomfort in any body part over the past 12 months led to rest, reduced activity, or leaving work [31]. The translation, localization, face validity assessment, and reliability of this questionnaire have been confirmed by Mokhtari Nia et al. [32].

### 2.5. Data analysis

All data were analyzed using SPSS v 24.0 software (IBM Corporation, Armonk, NY). The normality of the data was confirmed using the Shapiro-Wilk test. In the data analysis, due to the quantitative nature of the musculoskeletal disorder variables, mental workload, and work ability, the mean and standard deviation were used for their description. To examine the relationships between variables, Pearson correlation coefficient, post-hoc tests, and binary logistic regression were employed. A significance level set at 0.05 was considered in all analyses.

### 2.6. Ethical approval

This study is derived from a master’s thesis, the protocol for which was approved by the Research Council of the University of Social Welfare and Rehabilitation Sciences, Tehran, Iran (ethical code: IR.USWR.REC.1400.299). All participants provided written consent to participate in the study prior to its execution.

## 3. Results

In this study, the majority of participants were female (67.3%) and health workers (34.2%). Fifty-eight percent of the employees were married, and 86.6% of the subjects experienced a 7-hour workday (Table 2). The average of BMI, age, and work experience of the participants were determined to be 25 ± 3.73, 35.74 ± 7.52, and 11.63 ± 8.59 years, respectively.

**Table 2:** Demographic characteristics of participants.

Variable		Frequency	Precent
**Gender**	Female	133	67.3
	Men	64	32.7
**Age**	20-34	103	52.3
	35-44	62	31.5
	45-54	32	16.2
**Marital status**	Married	115	58.2
	Single	82	41.8
**Works Hour group**	7	171	86.6
	8	2	1
	12	24	12.4

Data analysis indicated significant relationships between dimensions of mental workload and work ability. In particular, the Frustration dimension had a significant inverse relationship with all dimensions of work ability (P < 0.05); such that an increase in mental workload, especially in the dimensions of current work ability compared with lifetime best, physical and psychological job needs, and sick leave during the past year, led to a decrease in feelings of frustration. This inverse relationship suggests that greater physical and mental ability leads to less feelings of frustration in participants. Conversely, there is a positive relationship between feelings of frustration and current illnesses; meaning that as the number of diagnosed illnesses increases, feelings of frustration also rise.

Furthermore, mental and cognitive workload also has a positive and significant relationship with No. of current diseases (P < 0.05). These findings may indicate the importance of managing mental and physical workload in workplaces. Moreover, the positive relationship between performance and efficiency with work ability in relation to physical and psychological job needs and cognitive and mental abilities suggests that improvements in job performance can lead to increased work ability under various conditions (Table 3).

**Table 3:** Correlation between mental workload dimensions and work ability dimensions.

Work Ability			Mental Workload dimension					
			Mental demand	Physical demand	Performance	Temporal demand	Effort	Frustration
	Mean±SD		66.24 ± 19.60	76.59 ± 15.13	76.01 ± 12.96	62.41 ± 15.80	67.66 ± 17.98	40.22 ± 32.02
**Current work ability compared with lifetime best**	7.03 ± 1.51	R	-0.113	**-0.155** ^*^	0.053	-0.098	0.027	**-0.591** ^**^
		P	0.114	**0.030**	0.460	0.171	0.707	**<0.001**
**Work ability in relation to demands of the job**	6.22 ± 1.22	R	**-0.201** ^**^	**-0.222** ^**^	**0.152** ^*^	**-0.183** ^**^	-0.124	**-0.558** ^**^
		P	**0.005**	**0.002**	**0.033**	**0.010**	0.083	**<0.001**
**No. of current diseases diagnosed by a physician and patient**	1.25 ± 0.95	R	**0.220** ^**^	-0.021	-0.133	**-0.171** ^*^	0.040	**0.376** ^**^
		P	**0.002**	0.772	0.063	**0.016**	0.577	**<0.001**
**Estimated work impairment due to diseases**	5.06 ± 1.20	R	-0.088	-0.004	0.073	0.028	0.034	**-0.416** ^**^
		P	0.217	0.956	0.310	0.692	0.639	**<0.001**
**Sick leave during the past year**	3.55 ± 0.62	R	**-0.296** ^**^	**-0.175** ^*^	0.015	-0.112	**-0.236** ^**^	**-0.367** ^**^
		P	**<0.001**	**0.014**	0.831	0.116	**0.001**	**<0.001**
**Personal prognosis of work ability 2 years from now**	4.60 ± 2.51	R	**-0.208** ^**^	-0.062	0.063	-0.053	0.013	**-0.637** ^**^
		P	**0.003**	0.388	0.379	0.464	0.856	**<0.001**
**Mental resources, referring to life in general, both at work and during leisure time**	6.95 ± 2.78	R	**-0.299** ^**^	-0.110	**0.249** ^**^	**-0.191** ^**^	**-0.176** ^*^	**-0.626** ^**^
		P	**<0.001**	0.123	**<0.001**	**0.007**	**0.014**	**<0.001**

**Correlation is significant at the 0.01 level,

*Correlation is significant at the 0.05 level

A comparison of findings between different groups showed no significant difference in work ability among various occupational groups (P > 0.05). However, occupational health experts had the highest (38.25 ± 7.29) and environmental health experts had the lowest (29.16 ± 6.5) levels of work ability among the job groups. Additionally, the comparison of mental workload among different occupational groups indicated a significant difference (P < 0.05). In this regard, environmental health experts had the highest (78.85 ± 7.68) and vaccinators had the lowest (50.87 ± 13.77) levels of mental workload among the job groups. Furthermore, the higher mental workload score was reported in individuals with longer working hours, and this increase was statistically significant. Moreover, there was a direct correlation between work experience and mental workload, which was statistically significant. An inverse significant relationship was also observed between work experience, age, and occupational ability. A direct and significant relationship was noted between mental workload and age, indicating that mental workload increases with age (Table 4).

**Table 4:** Correlation between different study variables.

Variables	Statistics		Qualitative			Quantitative					
	Mean±SD	Min-Max	Job	Gender	Works Hour Group	Work Experience		Age		Work Ability	
						R	P	R	P	R	P
**mental work load**	63.24 ± 13.26	21.67-89	**0.001**	0.230	0.043	0.225	**0.002**	1910	**0.007**	0.450	**0.001**
**work ability**	34.86 ± 7.61	19-45.5	0.317	0.190	0.530	-0.760	**0.001**	-0.747	**0.001**	–	–

The prevalence of WMSDs in the lumbar and other areas of the body among various occupational groups indicates a serious issue in occupational health. According to the results presented in Table 5, the highest prevalence is related to the lumbar region, with 63.5% for lifetime prevalence and 60.9% for annual prevalence. Furthermore, it has been determined that 29.4% of individuals have experienced lower back pain in a monthly period. In addition to the lumbar region, other areas of the body have also been affected, particularly the neck and knees, which have prevalence rates of 32% and 17.8%, respectively. These data indicate that WMSDs are not limited to the lumbar region but are also common in other areas such as the neck, shoulders, and legs (Table 5). Among occupational groups, the highest prevalence was estimated for nutrition experts (100%) and service personnel (86%).

**Table 5:** Prevalence of WMSDs in the extremities in the past 7 days and 12 months.

Variable	Acute Pain, Chronic Pain, or Discomfort							
	Lifetime Prevalence		Periodic Prevalence				Point Prevalence	
			Annual		Monthly			
	Frequency	percentage	Frequency	percentage	Frequency	percentage	Frequency	percentage
**Neck**	63	35	59	29.9	30	15.2	8	4.1
**Shoulders**	24	12.2	20	10.2	9	4.6	2	1
**Elbows**	11	5.6	11	5.6	4	2	1	0.5
**Wrists/hands**	6	3	5	2.5	1	0.5	–	–
**Upper Back**	31	15.7	30	15.2	13	6.6	3	1.5
**Low back**	125	**63.5**	120	**60.9**	58	**29.4**	19	**9.6**
**Hips/thighs**	25	12.7	24	12.2	11	5.6	5	2.5
**Knees**	35	17.8	33	16.8	10	1.5	4	2
**Ankles/feet**	29	14.7	27	13.7	18	1.9	9	4.6

Analyses indicate that WMSDs in different body regions exhibit distinct patterns based on age and sex groups. In the neck region, men aged 20–34 years showed a lower risk (Prevalence = 37%, OR = 0.46) compared to women (OR = 1.45). However, across the entire population, the risk of neck pain in the 20–34 age group was significantly higher (OR = 3.13, P = 0.021). For the lower back, men aged 35–44 years demonstrated a reduced risk (prevalence = 79.2%, OR = 0.64), while women aged 45–54 years exhibited a lower risk (Prevalence = 89.5%, OR = 0.63). Moreover, participants in the 45–54 age group reported the highest prevalence of lower back pain compared to other age groups. In contrast, no significant association was observed between sex and risk in areas such as the shoulders, elbows, and wrists/hands across age groups (P > 0.05). However, in the knee region, men aged 35–44 years had a reduced risk (OR = 0.53), whereas women aged 45–54 years showed the highest vulnerability (Prevalence = 36.8%). Results for the ankles/feet were non-significant, though the 20–34 age group displayed a trend toward increased risk across the population (OR = 2.65) (Table 6).

**Table 6:** Prevalence and odds ratios (OR) (95% CI) of WMSDs during the last 12 months in relation to age for males, females, and the whole population.

Age group (years)	Males			Females			Whole			P^**^
**Prevalence**	**OR**	**95% CI**	**Prevalence**	**OR**	**95% CI**	**Prevalence**	**OR**	**95% CI**	
**Neck:**										**0.023**
20–34	37	0.46	0.25–0.86	15.8	1.45	0.97–2.15	21.4	3.13	1.16–8.48	0.021
35–44	29.2	0.91	0.46–1.81	26.3	1.06	0.67–1.67	27.4	1.15	0.97–2.15	0.806
45–54	69.2	0.74	0.29–1.89	57.9	1.21	0.69–2.13	62.5	1.63	0.36–7.25	0.515
**Shoulders:**										**0.078**
20–34	18.5	0.47	0.23–0.97	6.6	1.53	0.81–2.87	9.7	3.23	0.85–12.18	0.072
35–44	8.3	0.76	0.27–2.13	5.3	1.24	0.46–3.37	6.5	1.63	0.21–12.46	0.632
45–54	23.1	0.77	0.30–1.96	15.8	1.23	0.52–2.90	18.8	1.60	0.27–9.53	0.604
**Upper Back:**										**0.108**
20–34	11.1	0.32	0.16–0.63	1.3	3.03	0.55–16.60	3.9	9.37	0.93–94.38	0.024
35–44	4.2	1.17	0.23–5.98	5.3	0.91	0.40–2.01	4.8	0.78	0.07–9.13	0.845
45–54	15.4	0.78	0.26–2.32	10.5	1.21	0.44–3.38	12.5	1.54	0.19–12.64	0.683
**Elbows:**										**0.716**
20–34	3.7	1.05	0.18–5.92	3.9	0.98	0.55–1.75	3.9	0.94	0.93–9.40	0.995
35–44	4.2	0.81	0.27–2.41	0	1.13	0.55–2.32	1.6	0.38	0.27–0.52	0.205
45–54	–		–	–	–	–	–	–	–	–
**Wrists/hands:**										**0.460**
20–34	14.8	0.76	0.32–1.81	10.5	1.12	0.74–1.70	11.7	1.48	0.41–5.37	0.551
35–44	12.5	1.51	0.54–4.18	21.1	0.81	0.53–1.24	17.7	0.54	0.13–2.26	0.391
45–54	7.7	3.36	0.52–21.58	31.6	0.61	0.37–0.98	21.9	0.18	0.02–1.73	0.108
**Low back:**										**0.029**
20–34	63	0.53	0.27–1.05	42.1	1.25	0.98–1.58	47.6	2.34	0.95–5.78	0.062
35–44	79.2*	0.64	0.28–1.46	65.8	1.27	0.86–1.87	71	1.97	0.60–6.51	0.258
45–54	76.9	1.62	0.68–3.86	89.5*	0.63	0.21–1.93	84.4*	0.39	0.06–2.76	0.337
**Hips/thighs:**										**0.925**
20–34	3.7	1.32	0.22–7.89	5.3	0.92	0.58–1.44	4.9	0.67	0.07–6.48	0.746
35–44	16.7	0.96	0.42–2.21	15.8	1.03	0.59–1.77	16.1	1.07	0.27–4.25	0.927
45–54	23.1	1.30	0.46–3.67	31.6	0.85	0.47–1.52	28.1	0.65	0.13–3.26	0.599
**Knees:**										**0.353**
20–34	11.1	0.96	0.34–2.66	10.5	1.02	0.69–1.49	10.7	1.06	0.26–4.33	0.933
35–44	33.3	0.53	0.29–0.96	13.2	1.75	0.86–3.58	21	3.30	0.93–11.71	0.057
45–54	15.4	2.15	0.59–7.85	36.8	0.67	0.39–1.13	28.1	0.31	0.53–1.83	0.185
**Ankles/feet:**										**0.324**
20–34	18.5	0.53	0.25–1.11	7.9	1.39	0.80–2.42	10.7	2.65	0.74–2.42	0.125
35–44	16.7	0.64	0.31–1.32	7.9	1.48	0.62–3.57	11.3	2.33	0.47–11.50	0.288
45–54	15.4	2.15	0.59–7.85	36.8	0.67	0.40–1.13	28.1	0.31	0.05–1.83	0.185

*Maximum prevalence of musculoskeletal disorders,

**Significance levels less than 0.05

Table 7 shows that the variable of work ability has a significant impact on WMSDs in the neck. With an increase of one unit in the work ability score, the likelihood of developing neck pain decreases by 13.5%; in other words, the higher the level of work ability, the lower the probability of experiencing neck pain. Furthermore, work ability also has a significant effect on WMSDs in the wrist area. For each one-unit increase in the work ability score, the likelihood of wrist disorders decreases by 8%. Additionally, this variable significantly affects WMSDs in the lumbar region, such that an increase of one unit in the work ability score reduces the likelihood of these disorders by 11.5%. Regarding pain in the hip/thigh area, it has been determined that the work ability score has a significant impact, and with an increase of one point in this variable, the incidence of hip/thigh pain decreases by 9%.

**Table 7:** Analyzing the effect of work ability and mental workload on WMSDs.

Location of the Trouble	Mental work load				Work ability			
	β	S. E	Exp(β)	P	β	S. E	Exp(β)	P
**Neck**	0.006	0.015	0.994	0.690	-0.144	0.027	0.865	**0.001**
**Shoulders**	0.018	0.021	1.02	0.380	0.064	0.033	0.938	0.052
**Elbows**	0.088	0.036	0.960	0.100	‒0.028	0.072	1.02	0.690
**Wrists/hands**	0.015	0.017	0.980	0.370	‒0.077	0.029	0.920	**0.009**
**Upper Back**	0.016	0.026	0.980	0.530	‒0.072	0.045	0.930	0.110
**Low back**	0.003	0.014	0.997	0.810	‒0.123	0.028	0.885	**0.001**
**Hips/thighs**	0.001	0.020	1.001	0.970	‒0.090	0.030	0.910	**0.005**
**Knees**	0.014	0.017	1.010	0.420	‒0.043	0.028	0.950	0.127
**Ankles/feet**	0.036	0.020	1.030	0.072	‒0.040	0.031	0.960	0.190

## 4. Discussion

This study provides a demographic profile of the health service workforce in a remote area, highlighting important characteristics relevant to occupational health. A notable finding is the gender distribution, with 67.3% of participants identifying as female, consistent with trends in healthcare that show higher female representation. This gender aspect reflects not only workforce composition but also broader social and economic factors influencing roles in healthcare. Similar observations have been noted in prior research, such as that by Buerhaus et al. (2017), which emphasizes the predominance of women in nursing. This demographic trend may relate to specific stressors encountered by female workers, particularly regarding work-life balance, a challenge particularly significant for the 58.2% of participants who are married [33]. The study found that 86.6% of participants work a standard 7 hours per day, aligning with typical expectations for healthcare roles. However, this contrasts with literature indicating increasing workloads and burnout among health professionals. For example, Aiken et al. (2002) noted that longer work hours are correlated with elevated stress and a higher prevalence of WMSDs.

[34]. The reported standard work hours may indicate a sustainable workload, but it raises concerns about the intensity of work during those hours and whether there are high mental demands. Participants had an average Body Mass Index (BMI) of 25 ± 3.73, classifying the workforce as overweight according to WHO standards. This contrasts with a study by Gifford (2015), which found higher obesity rates among healthcare workers in urban areas, suggesting that lifestyle factors or access to recreational activities may differ in remote settings [35]. There is a need for continuous health monitoring and interventions to promote healthier weight among health service employees, as excess weight increases the risk of both physical and mental health issues, exacerbating occupational stress. The average age of participants was 35.74 ± 7.52 years, with an average work experience of 11.63 ± 8.59 years, indicating a moderately experienced workforce. Research by Spector et al. (2007) suggests that while more experienced workers often report greater job satisfaction, they also experience higher stress levels due to established responsibilities. [36]. This nuanced relationship suggests that while years of experience might confer valuable skills and confidence, they may also bring about increased responsibilities that could exacerbate occupational stress, thereby highlighting the need for supportive environments and stress management strategies.

Data from Table 3 reveals important insights into the relationships between mental workload and work ability among health service workers. A significant finding is the inverse relationship between frustration, a dimension of mental workload, and various aspects of work ability. As frustration rises, workers report decreased current work ability compared to their peak performance, physical and psychological job demands, and increased sick leave (P < 0.05). This underscores the importance of understanding how psychological factors affect perceived work capabilities and highlights the role of mental wellness in occupational health. The inverse correlation suggests that workers who are physically and mentally capable experience less frustration, while feelings of frustration are linked to higher incidences of diagnosed illnesses, indicating a negative impact on mental well-being and work performance. This is consistent with existing research by Foster et al. (2018) and Shubayr et al. (2022), which found that higher workplace stress adversely affects job satisfaction and efficiency, emphasizing the need for proactive mental health management in the workplace [37,38].

The study indicates a positive relationship between performance, efficiency, and work ability concerning a job’s physical and psychological demands, suggesting that improving performance can enhance work ability. This aligns with Lee and Jo’s (2023) findings that job performance correlates with perceived job capabilities and employee well-being [39]. In healthcare, these relationships are crucial, as job strain can negatively impact service delivery and patient outcomes, particularly in underserved areas.

Interestingly, the study also found that mental and intellectual burdens significantly correlated with current illnesses (P < 0.05). This relationship implies that as workers face heavier cognitive demands, their physical health may decline, mirroring findings from studies such as that of Søvold et al., which illustrated how mental workload can exacerbate physical health conditions [40]. This delineates a clear pathway through which workplace stressors can translate into broader health issues, highlighting the importance of creating balanced workloads to mitigate these risks.

Moreover, when comparing these findings with studies conducted in urban settings, a notable similarity emerges in the pattern of relationships among mental workload, work ability, and health outcomes. Alzoubi et al. (2002) reported similar findings regarding the implications of mental strain among healthcare workers, emphasizing how stress correlates with burnout and job dissatisfaction [41]. However, a divergence can be noted in the demographic characteristics, as this current study primarily involved a workforce with a significantly higher proportion of women (67.3%), which may influence the study’s outcomes given that gender dynamics often play a role in workplace stress experiences and coping mechanisms, as discussed by Elez et al. (2021) [42].

The incidence of sick leave, which exhibited a significant correlation with the frustration dimension, confirms previous research conducted by Verhaeghe et al. (2003), indicating that elevated work-related stress is linked to absenteeism within healthcare environments [43]. This association highlights the necessity for workplace interventions designed to alleviate mental workload, thereby enhancing overall employee health and productivity, particularly in sectors that require substantial emotional and physical involvement, such as healthcare.

The results reveal a nuanced understanding of work ability and mental workload across different occupational groups within the healthcare sector. While there were no significant differences in work ability among the various groups (P > 0.05), a notable contrast emerged between occupational health and environmental health experts. Occupational health experts reported the highest professional skills rating at 38.25 ± 7.29, while environmental health professionals had the lowest at 29.16 ± 6.5. This discrepancy likely reflects the differing demands and challenges of these roles. Occupational health practitioners, who often employ multidisciplinary approaches to address workplace health issues, may develop greater adaptability and capability, as supported by similar findings in related research by Adamopoulos (2023) [44]. In contrast, the significantly higher mental workload reported among the environmental health experts (78.85 ± 7.68) compared to vaccinators (50.87 ± 13.77, P < 0.05) indicates acute stress characterized by the cognitive and emotional demands of their role, which may be traversing complex environmental assessments and regulatory frameworks. This increased mental workload is consistent with existing literature showing that positions requiring extensive documentation, planning and compliance tend to entail higher cognitive demands [45]. Interestingly, a key finding of this study is that mental workload was higher in individuals who worked more hours. This observation is confirmed by previous studies showing that longer working hours contribute significantly to psychological distress [46,47]. The evidence of a direct correlation between work experience and mental workload, combined with the finding that mental workload increases with age, presents critical implications for workforce management. With age commonly associated with accumulated demands, the need for tailored interventions becomes apparent, focusing on the development of resilience and management strategies for older employees to mitigate the potential increase in mental strain [48].

The study finds a significant inverse relationship between age, work experience, and perceived job ability, suggesting that older and more experienced workers may feel less capable in their roles. This aligns with Wright and Cropanzano’s (2000) research, which indicates that career progression can lead to a decline in self-efficacy due to changing job demands and expectations[49].

The analysis of mental workload in healthcare professionals reveals that they experience significant cognitive demands due to high responsibilities and regulatory pressures, which can lead to chronic stress and burnout, negatively affecting performance. However, the lack of significant differences in job abilities across various roles suggests a potential standardization of skills in health services [50]. This indicates a need for foundational training and skill sets that are applicable across the sector, highlighting the necessity for further research to explore how these capabilities can be collectively enhanced rather than being defined by specific roles.

The study highlights a significant public health challenge posed WMSDs, particularly low back pain, which has a lifetime prevalence of 63.5% and an annual prevalence of 60.9% among the workforce. With monthly prevalence rates at 29.4%, this underscores a chronic issue that necessitates urgent attention in occupational health strategies. According to the Global Burden of Disease, low back pain is a leading cause of disability globally, especially affecting those in physically demanding roles such as nursing and caregiving [51,52]. The prevalence rates found in this study align with previous research in healthcare settings; a meta-analysis by Şimşek et al. (2017) reported a prevalence of low back pain among health professionals ranging from 58% to 70% [53], indicating that the results of this study are consistent with established findings.

The prevalence of discomfort in areas such as the neck (32%) and knees (17.8%) alongside low back pain indicates a widespread issue of musculoskeletal disorders (MSDs), suggesting that the risk factors are systemic rather than localized. Research by Alruwaili et al. (2023) [54] highlights that healthcare workers often experience pain in multiple regions, reflecting the cumulative strain they endure. Notably, neck and shoulder complaints are common due to repetitive motions and poor ergonomic practices typical in healthcare settings.

The study found that nutritionists had a concerning 100% prevalence of WMSDs, while service workers reported an 86% prevalence. This disparity highlights the distinct demands of each occupation. Nutritionists often engage in prolonged counseling and manual food handling, which may lead to their high rates of musculoskeletal disorders. In contrast, service workers typically perform various physically demanding tasks, resulting in significant injury rates as well.

Notably, other body regions, such as the shoulders, elbows, wrists/hands, and thighs, reflected significant MSD concerns, however with lower prevalence rates. This multifaceted profile repeats findings from organizations like the National Institute for Occupational Safety and Health (NIOSH) which emphasizes the need for comprehensive ergonomic assessments across various job functions to mitigate risks of widespread injuries. The current results’ confirmation of the interconnectedness of WMSDs emphasizes the necessity for interdisciplinary strategies in management and prevention.

Furthermore, the results of this study integrate smoothly with the existing literature by showcasing how physical demands and ergonomic deficiencies contribute significantly to WMSD prevalence. Hamid et al. (2018) outlined how occupational factors such as manual handling, long hours in static positions, and inadequate breaks contribute to the development of WMSDs among healthcare employees [55]. The results suggest that workplaces need to reassess ergonomic standards and invest in preventative training and resources to alleviate injury rates.

The results presented in Table 7 reveal a significant relationship between occupational ability and the prevalence of WMSDs in various body regions, particularly the neck, wrist, low back, and hip/thigh areas. Notably, each one-unit increase in occupational ability score is associated with a decrease in the likelihood of developing neck pain by 13.5%, wrist disorders by 8%, low back disorders by 11.5%, and hip/thigh pain by 9%. These findings underscore the crucial role occupational ability plays in safeguarding health against chronic discomfort and injury, particularly within physically demanding job roles.

Enhancing occupational ability inversely affects the occurrence of musculoskeletal disorders (MSDs), aligning with ergonomic principles that improve job practices, physical health, and satisfaction. This is crucial in physically demanding sectors like healthcare and service industries, where boosting workers’ skills fosters productivity and well-being. Ziam et al. (2020) highlight the significance of ergonomic training in reducing neck and back pain among healthcare workers, underscoring the role of improved occupational competence in minimizing MSDs [56].

The study finds that mental workload does not significantly affect WMSDs in specific body regions like the shoulder, upper back, elbow, knee, ankle, or foot. This is surprising, as previous research often identifies mental workload as a key risk factor for physical strain, particularly in health professionals [57]. While mental workload impacts overall well-being and job performance, its direct connection to specific WMSDs is unclear, indicating a need for more detailed research. The study highlights the importance of investigating the conditions and mechanisms through which mental workload may physically manifest, noting that job stress can lead to long-term health issues such as burnout and absenteeism [58]. However, the lack of significant influence in this study might point toward the need for targeted investigations to explore the conditions under which mental workload begins to manifest physically. It may also highlight an opportunity to delve into the mechanisms through which mental workload exerts its influence on various regions of the body.

## 5. Conclusion

The findings of this research indicate that the work capacity of individuals in the occupational groups studied falls within an average range and has a significant relationship with WMSDs. Therefore, to improve working conditions, identifying factors that affect the reduction of work ability and implementing ergonomic measures should be prioritized. The overall mental workload in the examined occupational groups was found to be high, and there is also a significant relationship between mental workload and the prevalence of WMSDs. To reduce mental workload, corrective actions must be taken, ultimately leading to a decrease in employee disengagement and an increase in work ability, while also preventing the rising prevalence of WMSDs.

## Supporting information

S1 Data Final data used in the study. (XLSX)
